# Dental Jottings

**Published:** 1871-10

**Authors:** H. L. Sage


### DENTAL JOTTINGS.

BY H. L. SAGE.

### *Alveolar Abscess with Caries of the Superior Maxilla.*

In an article in the June number of the REGISTER for 1870, entitled "Alveolar Abscess with Caries or Necrosis," etc., reference was made on page 251, to a case of caries of the maxilla, then under treatment, with this remark: "the successful treatment of which, with the retention of the teeth included, I hope to be able to report at a future day." A successful result having been attained, the following may not be uninteresting. Perhaps those with more experience in the treatment of similar cases, might suggest some improvements in methods of practice; but that this was not a failure, is in some measure at least, a vindication of the course pursued, inasmuch as the cure was effected, without the sacrifice of the teeth.

Miss E. L., age 34; temperament bilio-sanguine; teeth long, dense, and yellow; teeth involved, the left superior central and lateral incisors Chronic case of seventeen years standing. Incidentally treated in connection therewith, the second left superior bicuspid.

The central incisor was filled in 1852, in the posterior proximal surface, and trouble was experienced soon after.

The patient stated that about two years ago, it was unsuccessfully treated for abscess through the root canal, it being a case "that baffled his skill," as the dentist expressed it, so that, at that time, it was a case of fifteen years standing. When first seen, the abscess discharged through a fistula in the gum, over the root of the central incisor. The affected teeth were remarkably firm, but somewhat more yellow and darker than those adjoining, and owing to their natural density, the excellence of the fillings, or both, were in a good state of preservation.

December 22, 1869. First treated the central incisor, by pumping creosote through the canal in the root, some of which appeared through the fistula in the gum. At the next sitting, after syringing out the cavity in the bone, the patient by snuffing detected a fluid in the nares. Perhaps, the nerve and artery passing through the naso palatine canal, had been destroyed by the encroachment of the disease (abscess) upon them, thus leaving the channel open for the passage of fluids—as an instrument inserted at the apex of the root of the central incisor, followed without obstruction a track, leading obliquely across the naso-palatine canal, making its exit on the right side of the median line of the hard palate, about twelve lines back of the right central incisor. The bone was rough and easily punctured. Soft parts on the lingual surface, were in a perfectly normal condition.

December 23. Syringed the cavity produced by the destruction of the bone, with a solution of the chloride of zinc; first through the root canal, and then through the fistula, which made its exit in either direction, showing the circuit to be unobstructed.

A broach introduced through the root, reached to the floor of the nares, and the patient located the pain produced, in that cavity. This indicated extensive destruction of bone.

In order to diagnose freely, enlarged the fistula, by stuffing it with cotton, saturated with iodine.

December 27. The treatment usual in cases of alveolar abscess, has been continued. Was enabled to observe the root plainly, which has sustained some loss. Removed from the cavity in the maxilla, the end of a broach and some cotton fibre, which was probably accidentally forced through the foramen of the root, during its treatment two years previously. The root was entirely bare on the labial surface, two thirds of its length from the apex, and on the inferior or lingual, at least one-eighth of an inch. Length of crown three-eighths of an inch, of root one-half of an inch.

December 30. Cut away the rough, dead, and broken down alveolar process around the borders of the chasm, dressing the inside with a strong solution of chloride of zinc, and the surface with tannin and glycerine.

January 3, 1870. Filled the root and crown cavity solidly with gold. Cut off, rounded and polished the end of the root. The advantage was here presented of observing the progress of the operation during the insertion of the filling at the apex, which was so compact, as to finish as smoothly, and polish as perfectly, as could be desired.

Amount of gold inserted, twenty-four grains.

About this time, and from the 4th to the 15th, at proper intervals, dressed the chasm with tinct. iodine, sometimes in combination with creosote, and the orifice with tannin and glycerine; but there was more pus present, than when the solution of chloride of zinc was used. For cleansing the cavity, syringed lightly with a wash composed of

℞.—Tinct. Iodine Comp., mxiv.
Acid. Carbolic Chryst., mvi.
Glycerina, ℥j. Aq. destillat, ℥v.*

Meanwhile the chasm was filling up slowly, and in the upper portion, under the alveolar plate, even with the borders of the perforation in the latter. Discovered a white powder

* From White's Dental Materia Medica.

on the folds of linen placed over the orifice of the chasm. Was it phosphate of lime, deposited from the pus? According to Paget, the pus from common ulcers contain but a trace of it, while that secreted from diseased bone, contains sometimes two and one-half per cent.

January 20. The chasm has filled up, to the extent of one-half its diameter. The inner surface has changed from a whitish or greyish appearance to red. The borders of the alveolar plate, and the portion of the cavity over the naso-palatine foramen are covered with cicatricial tissue. About the central incisor none of the alveolar process is bare. On removing a filling in the anterior approximal surface, of the adjoining lateral incisor, the tooth was found to be dead, owing, perhaps, to the death of the alveolar process surrounding it,—the latter, a consequence, doubtless, of the disease of the central incisor.

Dressed the cavity in the maxilla over the central incisor, with the sol. chlo. zinc—480 grs. to the ounce of water.

Filled the root and crown cavity of the lateral incisor, and continued the treatment, the patient calling the 22d, 26th and 29th.

February 1. One or two pits or sinuses were present, in the upper portion of the chasm, indicating the presence of carious bone.

February 17. Carried the fistula towards the left, by an incision, in order to diagnose more freely, enlarging the opening by stuffing it with cotton, saturated with tannin and glycerine.

February 21, Sufficient room having been obtained, examined and found a large extent of the alveolus over the root of the lateral incisor, rough, soft, and very easily broken down,—a drill entering to the depth of one-half of an inch, (six lines). There was a continuous loss of alveolar process, extending from the right central incisor to the left cuspidatus.

February 24. Owing to the enlargement of the fistula towards the left, that portion of the chasm over the central incisor, has lost its pointed shape, for the pus which came from the bone territory, about and above the lateral incisor root, had seemingly followed a channel leading from the apex of the latter, into the cancellous structure above, and across, and down into, and out of the fistula over the central incisor and laterally, through a sinus in the chasm above the latter. But a new channel having been formed for the pus, the old chasm assumed a healthful appearance.

End of the lateral incisor root bare, but not destroyed. Cut and scraped the borders and bottom of the chasm, bringing out granules of carious bone, though it was impossible, owing to the hemorrhage, to see the state of the parts during the operation. In order to control it, syringed with a solution of alum. It is evident that most of the trouble laterally, has proceeded from the presence of dead bone around or in the vicinity of the lateral incisor.

February 26. Filed off, smoothed and polished the end of the root of the lateral incisor, which was much further from the surface than that of the central. Length of root, one-half of an inch; of crown, three-eighths; of tooth, seven-eighths. Depth of chasm, including bone and soft parts, directly from the labial towards the lingual surface, one-half of an inch. Width over the lateral incisor, vertically, over one quarter of an inch; length, one half and one-sixteenth of an inch. Above the central incisor, it formerly extended under the alveolar plate to the floor of the nares.

February 28. Patient called. Dressed the abscess cavity.

March 2. The dressing of chlo. of zinc sol. caused considerable pain, and there was some swelling of the parts. To-day, a thick eschar lines the chasm. No pus. Syringed the cavity, and dressed lightly with iodine and creosote, which removed all pain.

March 8. Chasm has filled up one-eighth of an inch. Appearance white or grey. A short sinus points in the upper portion of the chasm, just under the incisive fossa. Dressing of sol. of chlo. of zinc, 40 to 60 grs. to ounce of water. Over the orifice, with tannin and glycerine. The root of the central incisor is being covered gradually.

March 12. No perceptible progress.

March 13. Looking better. Tinct. of iodine dressing. Shortened root of lateral incisor. Present length three-eighths of an inch. Orifice of chasm, diminished from side to side, three-sixteenths. Patient has commenced to take as a tonic, elixir phosphate, iron, quinine and strychnia. Reports good effects. Dresses surface of abscess daily with tannin and glycerine, and to cleanse it, syringes lightly. Have dressed from time to time according to the indications.

When using the sol. chlo. zinc, to form an eschar, to protect the granulating surface from the irritating effects of air and fluids, or substances foreign to the cavity, it usually remained from three to five or even seven days, and so long as this protection was afforded, there was little, if any, pus secreted from the surface. Though under this treatment the chasm filled up very fast, yet wherever it maintained a pointed shape, it seemed to be an indication of the presence of carious bone—a sinus always leading off from the pointed portion. These sinuses were followed up, from time to time, and dead bone found and removed, great caution being exercised.

March 19. There being a sinus at the bottom of the chasm, beneath the root of the lateral incisor, removed the new growth, and cut away bone in that direction; also, under and around the root of the lateral incisor, and between that and the root of the central. Removed ten or more pieces and granules of carious bone. In these operations, some of the bone being scraped away, was so fine, that its condition was undistinguishable, and it being impossible, owing to the hemorrhage, to obtain an inner view of the cavity; some healthy bone was, of course, necessarily taken away. To-day, after the operation, a view was for the first time secured of a portion of the bottom of the chasm, to the extent of about three lines in diameter of surface. A view is much more difficult to be had in the treatment of cases where teeth are remaining; access is not so easy, on account of the limited space in which to operate, and the manipulation is more delicate.

The portion of bone seen, was smooth, (having been scraped) white, and vascular, with two minute bleeding points, indicative of health. That removed was destitute of blood vessels, and its color, dark. In a line between the central and lateral incisor, a portion of the palatine plate was gone, the perforation being in diameter about three lines; an instrument inserted from the front, being plainly felt by the finger, if placed on the lingual surface, where, notwithstanding, the mucous membrane was healthy.

Patient called on the 21st, 23d and 26th,—the latter period being seven days since cutting. Over the bare bone, was an unorganized layer of greyish-white material.

The period of incubation over sawn or cut bone, according to Paget, lasts about ten days, before a change occurs; then an increased supply of blood; then the granulations commence to organize.

March 29. Sides encroaching upon each other rapidly. Chasm still pointed over the perforation in the palatine surface, which is overlapped with tissue from the sides.

April 5. Chlo. of zinc sol. formed no eschar the last time it was used. Seems to have a better effect when in combination with the surface dressing of tannin and glycerine.

April 15. Patient came late. Unsatisfactory view. Chasm filled nearly up to root of central incisor.

April 19 and 20. Light dressing of tinct. iodine.

April 25. Sinus remains at the bottom of the chasm. Soft parts very sensitive.

April 28. Smoothed off the borders of the perforation in the hard palate, and endeavored to make them as perpendicular as possible; also, cut towards the floor of the nares above the lateral incisor, but did not enlarge the orifice. Have used round hoes and Palmer burs with great advantage and convenience. Depth of chasm over five-eighths of an inch.

May 2. Granulating surface is greyish and "succulent, and abundantly supplied with blood." Syringed with calendula and water. Dressed with sol. chlo. of zinc.

May 3d and 4th. Patient also called.

May 6. Removed dead bone under the root of the lateral incisor, and towards the cuspidatus, near the palatine surface. Cut the borders well. Perforation in hard palate three-eighths of an inch in diameter. The lateral incisor root overhangs the chasm over one-eighth of an inch.

May 7, 12, 16 and 19. Dressed according to the indications. Root of lateral incisor is being covered on the labial surface. Shall aim to induce rapid closure, and if hereafter an operation becomes necessary, cut from the lingual surface.

May 21. Examined the left superior second bicuspid, which the patient stated, had been treated through the root, for abscess, two years ago, and pronounced cured; the fistula in the gum having closed. It was then an abscess of three years standing. Thinking that, possibly, some connection might exist between this case and the one under treatment, by means of a sinus, leading from the root of the second bicuspid, through the spongy bone, back of, and past the roots of the first bicuspid and cuspidatus, near the lingual surface,—into the chasm, (but which I have never fully determined,) proceeded to make an examination. First removed the crown filling of sponge gold, in the posterior approximal surface, and the root filling of cotton. Filled the root canal temporarily, with floss silk saturated with creosote, and the crown cavity with oxychloride of zinc.

May 23. Gum had swelled over the root. This question then occurred: has the bicuspid been diseased during these two years? The fistula in the gum being closed, did disease still remain, the contents of the abscess emptying into the chasm over the incisors, through the sinus in the spongy bone above referred to?

Made application to the gum to await further developments.

May 25. Much pus over the root, which flowed freely after lancing the gum. Bone hard, and somewhat sensitive, but absorption enough to permit the easy passage of a drill. Introduced floss silk and creosote into the abscess through the fistula.

May 28. Removed temporary filling. Inserted point of hypodermic syringe, loaded with a suitable quantity of creosote, into the root canal, sealed up with plaster, and forced the medicine through. It appeared through the gum freely. Refilled the root temporarily. Introduced cotton saturated with tinct. iodine in the orifice of the fistula. Third dressing. Tested the state of the first bicuspid and cuspidatus adjoining, by means of ice. Found sensitiveness to contact—indicating life; the teeth under treatment not responding.

June 18, 1870. Fistula entirely healed. Gum healthy. Afterwards inserted a permanent filling, though the root foramen was much enlarged by the destruction of the apex.

To continue the report of the particular case under treatment—May 25 and 28, and June 2 and 6, dressed as usual.

June 18. Having returned from a visit to the country, the patient again called. Abscess cavity filled up very much; root of lateral incisor is being arched over with tissue. Perhaps the treatment of the second bicuspid may have had a favorable effect upon the case. No sinus remains in any visible portion of the cavity. Dressed with sol. chlo. zinc.

June 23 to 30. Same dressing; sixty grs. to ounce of water, and gums with tannin and glycerine. The patient being about to leave town for a season, prescribed mild dressings with syringe.

July 29. Returned. Same progress.

August 3. A good view of all parts of the much reduced chasm, excepting in the upper posterior corner, which is arched over.

August 5. Dressing of tinct. iodine and tannin and glycerine. Frequent dressings are best, when mild.

August 9, 16 and 17. Light dressings.

August 20. At the suggestion of Dr. Atkinson, dressed the chasm with the elixir of vitriol, (aromatic sulph. acid,) and to keep the "pocket" from being filled up, or encroached upon by the lip, placed a thin strip of hollywood or a properly molded piece of gutta percha plate over it.

August 22. The apex of the root of the central incisor, being very near the surface, rendering the process of covering with tissue very slow, cut down, made flat, and smoothed it off on the labial surface, in order to make room for a *thicker*, and thus induce a more *rapid* growth. The roots of these teeth, have a constant tendency to approach the surface. The contraction of the cicatrix at the bottom of the chasm, probably has this effect. No pus. "Pocket" filled with an eschar, produced by the elixir of vitriol.

August 25 and 27. Eschar not entirely removed.

August 30. Deepest portion of pocket filled up one-half since the application of sulph. acid, though the size had previously been greatly reduced.

September 3. Dressed with the elixir of vitriol.

September 10. Eschar remains in deepest portion of chasm. Scraped the lips (of chasm) to induce union by adhesive inflammation.

September 14. Eschar not removed.

September 22, 24 and 26. Scraped the lips, or dressed with tinct. iodine lightly.

November 5. Patient has been stimulating the surface with a dressing of essence of peppermint for a few days past. Progress good. Roughed, scratched, or lightly scraped the lips of the pocket.

January 13, 1871. Depth of chasm reduced to about one line—length two lines. Just the smallest portion of the apex of the root of the central and lateral incisor is visible. Has received no dressing for several weeks.

July 19. The patient having been absent, have not seen the case since January 13. Root of central incisor, entirely covered by a mere film of healthy tissue. Root of lateral incisor, completely enveloped by a new growth. Gums about the cervical portions of the teeth, very hard, tough and healthy, with very little feeling. The root of the central incisor being so near the surface, was the last to receive the covering. The lips of the chasm, pocket, or cavity, united in the middle, and from that point approached and covered the root of the central incisor. There is just the slightest depression where the loss occurred—nothing indicating disease

The central incisor is as firm as its mate, and the lateral has nearly as much firmness, though the alveolus around it was destroyed more extensively.

As the chasm in the maxilla, reached at one time, in depth over one-half of an inch; in length, from side to side, five-eighths, extending in width to the floor of the nares, the production of tissue is nearly as extensive as was the loss—making allowance for the contraction of cicatricial tissue, which is visible to some extent, on the palatine surface, in the region of the loss, but which is constantly and rapidly thickening over the roots on the labial surface.

